# A Comparative Study on the Multidimensional Features of Hereditary and Sporadic Medullary Thyroid Carcinoma Patients: A Single-Center Retrospective Study

**DOI:** 10.3390/medicina61071164

**Published:** 2025-06-27

**Authors:** Muzaffer Serdar Deniz, Narin Nasiroglu Imga, Belma Ozlem Tural Balsak, Asiye Safak Bulut, Furkan Savas, Busranur Cavdarli, Cevdet Aydin, Oya Topaloglu, Reyhan Ersoy, Bekir Cakir

**Affiliations:** 1 Department of Endocrinology and Metabolism Diseases, Ankara Bilkent City Hospital, Ankara 06800, Turkey; narinnasiroglu@gmail.com (N.N.I.); belmabalsak@gmail.com (B.O.T.B.); 2 Deparment of Pathology, Ankara Bilkent City Hospital, Ankara 06800, Turkey; asafakbulut@gmail.com; 3 Department of General Surgery, Breast and Endocrine Surgery Clinic, Ankara Bilkent City Hospital, Ankara 06800, Turkey; opdrfurkansavas@gmail.com; 4 Deparment of Medical Genetic, Faculty of Medicine, Ankara Yildirim Beyazit University, Ankara 06800, Turkey; bcavdarli@gmail.com; 5 Deparment of Endocrinology and Metabolic Diseases, Faculty of Medicine, Ankara Yildirim Beyazit University, Ankara 06800, Turkey; cevdetaydin68@hotmail.com (C.A.); oyasude@gmail.com (O.T.); reyhanersoy@hotmail.com (R.E.); bcakir@ybu.edu.tr (B.C.)

**Keywords:** medullary thyroid carcinoma, sporadic, hereditary, RET mutation, germline

## Abstract

*Background and Objectives:* Medullary thyroid carcinoma is a rare neuroendocrine malignancy, with sporadic and hereditary forms accounting for 75% and 25% of cases, respectively. This study compares the clinicopathological features of sporadic medullary thyroid carcinoma (sMTC) and hereditary medullary thyroid carcinoma (hMTC) using real-world data to provide risk factors that aid in the early detection of the disease. *Materials and Methods:* The retrospective study comprised 77 patients with confirmed MTC treated at a tertiary referral center between January 2019 and December 2024. Patients were classified as hMTC (*n* = 11) or sMTC (*n* = 66) based on RET proto-oncogene (RET) genetic testing, whereas harboring a germline RET mutation indicated hMTC. Demographic, clinical, laboratory, radiological, histopathological, and genetic data were collected. *Results:* hMTC patients were significantly younger at diagnosis, with a comparable gender distribution (*p* = 0.738), and more often had a previous case of MTC within the family history. Pheochromocytoma occurred exclusively in hMTC. Multicentric tumors were more frequent in hMTC, and non-diagnostic Bethesda I cytology was higher in hMTC. *Conclusions:* While confirming established differences, this study provides detailed pre-operative diagnostic parameters and surgical approaches that can guide clinical decision-making in resource-limited settings where genetic testing may not be immediately available.

## 1. Introduction

Medullary thyroid carcinoma (MTC) is a rare form of thyroid cancer originating from the parafollicular C cells of the thyroid gland. The incidence of MTC is relatively low, with approximately one to two new cases per million people annually [1,2]. It accounts for approximately 2–4% of all thyroid cancer cases but is responsible for 8–15% of mortality from thyroid malignancies [3,4]. MTC is a neuroendocrine tumor able to secrete calcitonin and other peptides normally secreted by parafollicular C cells, such as CEA [5]. Suspicious palpable thyroid nodules were detected on physical examination [2,6,7,8]. MTC diagnosis relies on elevated serum calcitonin and CEA, fine-needle aspiration with calcitonin washout, and confirmation by histopathology (amyloid deposition, Congo-red positivity) with immunostaining for calcitonin [2,7,8], even though calcitonin-negative cases are present [9]. Further genetic analysis, specifically of RET and RAS mutations, identifies MTCs [6,7].

MTC can be classified into sporadic and hereditary cases [1,10]. Sporadic MTC (sMTC) is more prevalent, comprising about 75% of all MTC cases defined by the lack of family history of MTC and inherited RET mutation [1,10]. Sporadic cases with an RET mutation presented with a poorer prognosis, and more severe pathology resulting in worse patient outcomes [11,12]. Hereditary MTC (hMTC) accounts for the remaining 25% of cases and is associated with an inherited autosomal dominant trait, particularly in the RET proto-oncogene [12,13,14]. This form often presents as part of multiple endocrine neoplasia type 2 (MEN2) syndromes [15,16], which may include other endocrine tumors such as pheochromocytoma [5,15] and hyperparathyroidism [5]. Apart from the genetics of sMTC and hMTC, it is possible to differentiate between them case by case as they differ in their clinical presentation, age of onset, and tumor characteristics. hMTC typically presents at a younger age (often in childhood or early adulthood), while sMTC is usually diagnosed later in life [17,18,19,20,21]. hMTC is much more likely to be multifocal and bilateral (up to 81% bilateral in hMTC vs. 27% in sMTC) [19,20,21,22], whereas sMTC often presents with larger tumors and at a more advanced stage (stage III/IV in 73–80% of pediatric sMTC vs. 28% in hMTC) associated with lymphatic spread [20,22]. Serological markers calcitonin and CEA are more efficient in identifying sMTC diagnosis compared to hMTC [20,23]. While all the factors outlined above are essential for an accurate diagnosis, studies found that survival outcomes for matched stage sMTC and hMTC are comparable, suggesting similar tumor behavior once the stage is accounted for [17,19,22]. This highlights the importance of early detection and genetic screening in clinical management. However, comprehensive real-world comparative data on pre-operative sonographic features, cytopathological findings, and detailed surgical management approaches remain limited, particularly from Turkish tertiary centers.

Despite extensive knowledge of sMTC and hMTC molecular characteristics, significant gaps remain in integrating these findings into routine clinical practice. Since RET mutation status significantly affects patient outcomes, real-world comparative data are crucial for optimizing diagnostic strategies, risk stratification, and surgical planning. Our study addresses this need by comparing the clinicopathological characteristics of hereditary and sporadic MTC patients at a tertiary referral center. The aim was to compare the clinical, pathological, sonographic, and molecular features of hereditary and sporadic medullary thyroid carcinoma in a Turkish tertiary center cohort to guide genetic counseling and improve patient outcomes.

## 2. Materials and Methods

### 2.1. Study Design and Setting

This cross-sectional comparative study was conducted with patients who had been diagnosed with MTC based on histopathological examination. They presented to the Endocrinology and Metabolic Diseases Clinic of Ankara Bilkent City Hospital between January 2019 and December 2024, were evaluated by the multidisciplinary endocrinology council, and were referred for thyroid surgery. The research was approved by the Ankara Bilkent City Hospital Medical Research Scientific and Ethical Evaluation Board No. 1 (approval date: 12 March 2025, number: 1/1119/2025) and is being carried out in compliance with the Helsinki Declaration’s ethical criteria and the Strengthening the Reporting of Observational Studies in Epidemiology (STROBE) guidelines.

### 2.2. Study Population

The study was conducted in an adult endocrinology clinic, which excluded pediatric and adolescent patients who constitute a significant portion of hereditary MTC cases. This may have contributed to the lower-than-expected hereditary MTC prevalence in our cohort. Patients who (a) were 18 years old or older, (b) had a confirmed histopathological diagnosis of MTC, and (c) had a complete record of demographic, pathological, laboratory, radiological, and genetic data were included in the study. Patients (a) who were pregnant or in the lactation period; (b) whose records, including demographic, pathological, laboratory, radiological, or genetic data, were incomplete; or (c) who had immunodeficiency were excluded from the study.

#### Sporadic vs. Hereditary Stratification

A total of 77 patients were included in the study, stratified as sMTC or hMTC according to the revised ATA guidelines [7]. In brief, MTC patients were tested for germline mutations in the RET proto-oncogene, targeting exons 8, 10, 11, 13, 14, 15, 16, and 19. Patients with a germline RET mutation were classified as hMTC, consistent with clinical entities such as multiple endocrine neoplasia type 2A (MEN 2A), type 2B (MEN 2B), or familial medullary thyroid carcinoma (FMTC), whereas patients without germline RET mutations were classified as sMTC [10,14]. Somatic RET mutation analysis from tumor tissue was not routinely performed during the study period and therefore could not be included in this analysis. Among 77 index nodules, 3 had Bethesda II and 3 had Bethesda III cytology, all later confirmed as medullary thyroid carcinoma.

### 2.3. Data and Variables

Patients’ clinical, demographic, and genetic data were collected. The history of thyroid carcinoma was evaluated within the primary relatives. Both anatomical and pathologic staging of MTC cases were carried out according to the American Joint Committee on Cancer Staging Manual [24].

Thyroid volume was calculated according to the formula:

Volume = π/6 × Length (cm) × Width (cm) × Depth (cm)

Thyroid weight was determined by weighing the pathology material.

All the serological parameters were collected from routine tests needed at the pre-operative stage.

Germline RET mutations were analyzed from the pre-operative peripheral blood samples. DNA was extracted from peripheral blood and sequenced by next-generation sequencing (MSeq, Illumina, San Diego, CA, USA) within known variants listed in CDHS-18351Z-8 (https://www.qagen.com). The raw data were further analyzed at Ankara Bilkent City Hospital Medical Genetics Department with https://apps.tr.qiagenbioinformatics.com (accessed on 23 June 2025) GRCh37 (h19) as a reference genome. The variants were evaluated according to ACMG Standards and Guidelines 2015 [25].

### 2.4. Surgical Management

The surgical approach was determined according to the 2015 American Thyroid Association guidelines for medullary thyroid carcinoma. Central lymph node dissection was performed based on pre-operative risk assessment and intra-operative findings. Lateral neck dissection was indicated when pre-operative imaging showed suspicious lymph nodes confirmed by fine-needle aspiration biopsy with elevated calcitonin washout levels. In three cases with benign pre-operative cytology, only total thyroidectomy was initially performed.

Post-operative follow-up included serial calcitonin and CEA measurements, with imaging studies as clinically indicated.

### 2.5. Statistical Analysis

Because we intended to include all eligible patients, no sample size was calculated before the study. Statistical analyses were performed using SPSS version 23 (IBM Corp. in Armonk, NY, USA). The distributions of numerical variables were evaluated using the Shapiro–Wilk and Kolmogorov–Smirnov tests. Descriptive statistics were presented as frequency (*n*) and percentage (%) for categorical variables, and median with an interquartile range (IQR) for non-normally distributed numerical variables. Continuous numeric data were analyzed using the Mann–Whitney U test. Categorical data were analyzed using the Pearson Chi-square test or Fisher’s exact test. *p* < 0.05 was accepted as the statistical significance level.

Post hoc power analysis was conducted using PASS 11 to evaluate the statistical power of our study design. For the primary distinguishing features between hereditary and sporadic MTC, our sample sizes of *n* = 11 and *n* = 66 provided 99% power to detect the observed difference in family history of thyroid cancer (63.6% vs. 3.0%) and 91% power to detect the difference in tumor multicentricity (36.4% vs. 3.0%) at α = 0.05 (two-tailed Fisher’s exact test).

## 3. Results

**Figure 1:**
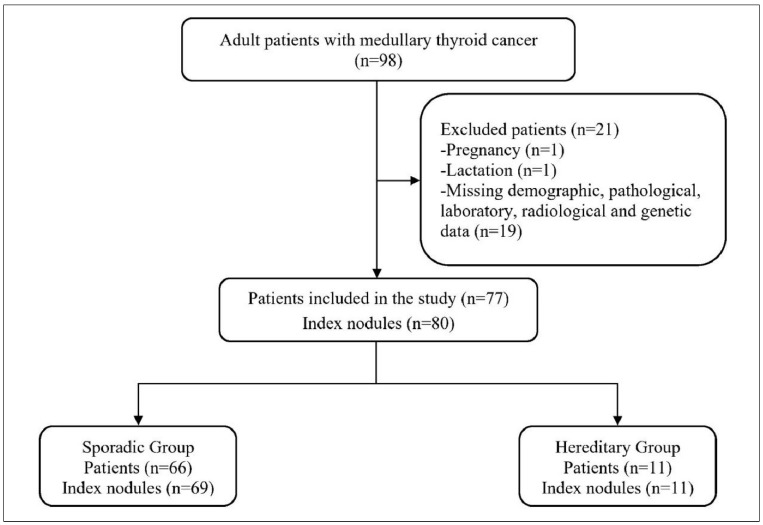
Flow diagram of the study.

A total of 77 patients with histopathologically confirmed MTC were included—66 in the sporadic group and 11 in the hereditary group (Figure 1).

The genetic tests showed that in the hereditary group, RET mutations were identified in 10 of 11 patients (90.9%), predominantly c.1901G>A (p.Cys634Tyr) and c.2410G>A (p.Val804Met) pathogenic variants; a minority harbored variants of uncertain significance (Table S1). The hereditary cohort was significantly younger and had a markedly higher rate of positive family history of thyroid cancer compared to sporadic cases. The thyroid function along with serum levels of free T_3_, free T_4_, TSH, thyroglobulin, anti-TG, anti-TPO, calcitonin, CEA, thyroglobulin, and family history of other tumors were comparable between the two study groups (*p* > 0.05 for all) (Table 1).

Histopathologic analysis demonstrated a similar median tumor size (13.0 mm in both groups; *p* = 0.749). Although multicentricity was significantly more frequent in hereditary MTC (36.4% vs. 3.0%; *p* = 0.003), only 36% of the hereditary and 3% of the sporadic group presented. Rates of extrathyroidal extension, vascular invasion, and surgical margin positivity did not differ significantly (*p* > 0.05) (Table 2).

TNM staging distributions were similar, though a higher proportion of hereditary cases presented with lateral lymph node involvement (T^1b^N^1b^M^0^: 18.2% vs. 6.1%; T^2^N^1b^M^0^: 18.2% vs. 10.6%). Anatomical and pathological stage groupings showed no statistically significant differences (Table 3).

Ultrasound features of the index nodules, including volume, longitudinal diameter, echogenicity, calcification pattern, and EU-TIRADS score were comparable across groups (all *p* > 0.05). However, Bethesda category I (non-diagnostic) cytology was more common in hereditary cases (27.3% vs. 0.0%; *p* = 0.018). Concurrent papillary thyroid carcinoma was uncommon and similarly distributed (Table 4).

Concurrent papillary thyroid carcinoma (PTC) was identified in seventeen patients from the sMTC group and in one patient from the hereditary group. There were no statistically significant differences between the groups regarding the distribution of PTC variants (*p* = 0.999). The median PTC diameter was slightly larger in the sporadic group (7.0 mm) compared to the hereditary group (1.0 mm), though this was not statistically significant (*p* = 0.111) (Table 5).

Post-operative response to therapy was evaluated in all 77 patients according to ATA guidelines. Forty-five patients (58.4%) demonstrated an excellent response, fourteen (18.2%) had structural disease, thirteen (16.9%) showed biochemical incomplete response, and five (6.5%) had indeterminate response.

## 4. Discussion

We collected demographic, clinical, and genetic data from 77 MTC patients at a tertiary referral center over five years. We stratified those 77 cases into hMTC vs. sMTC by the presence of germline RET mutation and correlated the clinical, demographic, and histopathological findings to facilitate prognosis. Our findings majorly showed similarities that sMTC and hMTC shared in the context of gender thyroid function, serological markers, histopathological specifics, and clinical features of index nodules. However, our findings indicate that a family history of MTC, the presence of pheochromocytoma, and multicentric tumors are key factors in distinguishing hereditary from sporadic forms of MTC. These red flags combined with genetic testing might help identify MTCs, particularly hMTCs which were presented two decades earlier than sMTCs, in the early stages and improve patient outcomes. Our study contributes to the literature by providing detailed pre-operative imaging and cytological data that may assist clinicians in risk stratification prior to genetic testing results, particularly relevant in healthcare systems with limited genetic testing availability.

In our cohort, 14.3% were hMTC, and 85.7% presented with sMTC, a rather enlarged population of sMTC compared to the published studies showing 75% [22,26]. The hMTC was represented earlier in life [20,23]. A study from the USA, consisting of a cohort of 144 patients younger than 21 years old, showed that the majority (*n* = 124, 86%) of the patients harbored hMTC and only a small proportion presented sMTC (*n* = 20, 14%) [23]. The patients with sMTC were also significantly older than the hMTC patients, 19.0 vs. 13.0, respectively [23]. Although the distribution of overall sporadic vs. hereditary MTC cases is comparable with the literature, the reduced hMTC population might account for the study inclusion criteria wherein the children and adolescents were excluded.

The Bethesda System for Reporting Thyroid Cytopathology provides standardized diagnostic categories, with categories II (benign) and III (atypia of undetermined significance) representing the most controversial classifications in terms of malignancy risk. In our cohort, Bethesda category II was identified in three nodules (4.3%) from the sporadic group, while category III was found in three nodules (4.3%) also from the sporadic group. All cases ultimately diagnosed as MTC came from categories V and VI, consistent with the high malignancy rates associated with these categories. Bethesda category II nodules carry a malignancy risk of approximately 5–15%, while category III nodules have a risk of 10–30% [27]. In our cohort, all such nodules were ultimately confirmed as MTC, underscoring the limitations of cytology in detecting this tumor subtype.

Calcitonin released from the parafollicular C cells and its elevated level are often associated with MTC [7,8]. Studies have shown that calcitonin is a valuable indicator of MTC [28], even in the absence of enlarged nodules [29]. Increased post-operative calcitonin levels are also linked with lymphovascular invasion and lymph node metastasis in MTC [30]; however, there are a limited number of studies comparing calcitonin levels in sMTC vs. hMTC. Raue et al. focused on the survival of sMTC vs. hMTC patients and used RET mutation to classify hMTC and elevated serum calcitonin levels for sMTC [17]. In our study, pre-operative serum calcitonin levels were comparable between sMTC (217.5 pg/mL (52.7–858.0)) and hMTC (223.0 pg/mL (21.0–3202.0)) (*p* > 0.05). In another study conducted with a Japanese cohort which had a similar sporadic vs. hereditary MTC distribution to our study, calcitonin levels were similar (*p* > 0.05) between the two types [21]. Although many studies emphasize the importance of both pre-operative and post-operative calcitonin levels, calcitonin is not a golden standard, as diverse types of thyroid malignancies cause an elevation in calcitonin levels [31] and, although rare, calcitonin-negative MTCs exist [9].

Inherited gain-of-function RET mutations are strongly correlated with hMTC [13]. Our results showed that 62% of the hMTC cases had a first-degree relative who also had MTC, which is a rather lower percentage compared to the 100% within the current literature [26]. Inherited RET mutations give rise to MEN2, a condition in which benign and malignant endocrine neoplasms co-exist, such as MTC combined with pheochromocytoma or hyperparathyroidism [15]. Coincidentally, germline RET mutations are associated with multicentric or bilateral tumors and secondary benign endocrine conditions; 50% of both MEN2A and MEN2B cases presented with pheochromocytoma [15,21,22]. Our findings determined that only 36.4% of hMTC and 3% of sMTC cases had multicentric tumors. Although tumor multicentricity significantly distinguishes hMTC from sMTC (*p* = 0.003), its evaluation on a case-by-case basis remains difficult. Notably, Machens et al. showed that when sMTC possesses multicentric tumors, it is often associated with more aggressive features such as lymphatic invasion, extrathyroid extension, higher rates of lymph node and distant metastases, and lower rates of recovery [19]. The significantly higher rate of non-diagnostic cytology in hereditary cases has important surgical implications. When fine-needle aspiration biopsy is inconclusive, surgeons may need to rely more heavily on clinical suspicion and genetic testing results for operative planning. This finding suggests that alternative diagnostic approaches, such as calcitonin washout from fine-needle aspiration, may be particularly valuable in suspected hereditary cases where initial cytology is non-diagnostic.

Histopathologic examination alone is insufficient to stratify sporadic from hereditary MTC, because histopathologic specifications such as multicentricity, bilaterality, desmoplastic stroma, and C cell hyperplasia are not exclusive to one type [32,33], though they are more frequent in hereditary cases [30,34]. Our findings also provide the same trend in which, except for multicentricity, the histopathological features were comparable between sMTC and hMTC (*p* > 0.05). Clinical presentation and histopathology cannot reliably exclude hereditary disease. Clinical manifestations, histopathological findings, biochemical analysis, and genetic testing must complement each other to define a healthy and accurate diagnosis and patient management. The significantly higher rate of non-diagnostic (Bethesda I) cytology in hereditary cases (27.3% vs. 0%) is noteworthy and may reflect the smaller size or different tissue characteristics of hereditary tumors. This finding emphasizes the importance of genetic testing in suspected hereditary cases, even when initial cytology is non-diagnostic.

Our findings emphasize the importance of integrating clinical suspicion with genetic confirmation. Although features such as early-onset disease, multicentric tumors, and pheochromocytoma may point toward hereditary MTC, they are not exclusive to sporadic or hereditary MTC. Therefore, a combinatory approach with genetic testing for RET mutations remains the most reliable tool for stratifying MTC patients. Early identification of hMTC through germline analysis not only allows for timely and potentially curative surgery but also enables cascade screening in at-risk family members, offering an opportunity for prophylactic intervention before malignancy develops. Moving forward, incorporating routine RET analysis, even in sporadic cases, and expanding molecular profiling to include somatic mutations in sMTC may refine risk assessment and management strategies. Our genetic findings emphasize that all MTC patients should undergo RET testing, regardless of family history. While most of our hereditary cases had a positive family history of thyroid cancer, over one-third presented without obvious familial clustering. This highlights that negative family history does not exclude hereditary disease and supports universal genetic testing recommendations in MTC patients.

Our study has a retrospective, single-center design, comprising a marginally smaller hMTC population (*n* = 11). A key limitation is the exclusion of pediatric and adolescent patients, who represent a substantial proportion of hereditary MTC cases. This may have contributed to the lower-than-expected hMTC prevalence (14%) in our cohort. While our hereditary MTC group was relatively small (*n* = 11), post hoc power analysis confirmed excellent statistical power (>90%) for detecting the clinically meaningful differences observed in family history and tumor multicentricity, validating the robustness of our key findings. Our follow-up period was relatively short, which limits our understanding of long-term outcomes, as MTC can recur many years after initial treatment. The exclusion of pediatric and adolescent patients represents a significant limitation, as hereditary MTC often presents earlier in life and early intervention is crucial in MEN2 syndromes. The absence of somatic RET mutation data from tumor samples represents another limitation, as these mutations may provide additional prognostic information in sporadic cases. Additionally, although the germline mutations were analyzed from the peripheral blood of the patients, somatic mutations for the sMTC group from tumor samples were not studied. The approach of this article makes it one of the few representing the comparison of clinical and histopathological features of sporadic vs. hereditary MTC; the lack of somatic mutation data reduces the impact of the analysis.

## 5. Conclusions

In conclusion, while sMTC and hMTC share several clinical and pathological characteristics, specific features and genetic profiling are essential for accurate classification and management. Our findings reinforce the value of comprehensive diagnostic strategies, but should be interpreted cautiously given the limited outcome data. Future multicenter studies including pediatric populations are needed to provide more comprehensive comparative data. These results provide practical guidance for surgeons and genetic counselors, especially regarding the importance of genetic testing in younger patients with multiple tumors or pheochromocytoma, even when family history appears negative.

## Figures and Tables

**Table 1:** Demographics, clinical features, and laboratory findings of the patients.

Variables	Sporadic Group (*n* = 66)	Hereditary Group (*n* = 11)	*p*
Age (year), Median (IQR)	55.0 (47.0–64.0)	35.0 (28.0–56.0)	0.021
Gender, *n* (%)			
Male	24 (36.4)	5 (45.5)	0.738 ^a^
Female	42 (63.6)	6 (54.5)	
Family history of thyroid cancer, *n* (%)	2 (3.0)	7 (63.6)	**<0.001**
Thyroid function status, *n* (%)			
Hyperthyroidism	3 (4.5)	0 (0.0)	0.999 ^a^
Hypothyroidism	7 (10.6)	1 (9.1)	
Euthyroidism	56 (84.8)	10 (90.9)	
Family history of other tumors *n* (%)	6 (9.1)	3 (27.2)	0.082
Pheochromocytoma, *n* (%)	0 (0.0)	2 (18.2)	**0.019 ^a^**
Free T_3_ (pg/mL), Median (IQR)	3.35 (3.13–3.71)	3.55 (3.18–4.05)	0.095
Free T_4_ (pg/mL), Median (IQR)	1.16 (1.08–1.26)	1.23 (0.95–1.50)	0.620
TSH (μIU/mL), Median (IQR)	1.49 (1.05–2.60)	1.90 (0.61–2.46)	0.959
Thyroglobulin (ng/mL), Median (IQR)	14.5 (6.1–40.5)	15.0 (6.2–26.0)	0.808
Anti-TG (IU/mL), Median (IQR)	2.20 (0.90–15.55)	1.00 (0.60–1.30)	0.081
Anti-TPO (IU/mL), Median (IQR)	41.0 (28.0–68.2)	62.0 (28.0–65.0)	0.575
Calcitonin (pg/mL), Median (IQR)	217.5 (52.7–858.0)	223.0 (21.0–3202.0)	0.838
CEA (ng/mL), Median (IQR)	5.30 (1.84–28.42)	3.10 (1.49–42.70)	0.738
Type of surgery, *n* (%)			
TT	19 (28.8)	1 (9.1)	0.137 ^a^
TT + CLND	26 (39.4)	3 (27.3)	
TT + CLND + LLND	21 (31.8)	7 (63.6)	

^a^ Mann–Whitney U test was used. Note: IQR: inter-quartile range; T_3_: triiodothyronine; T_4_: thyroxine; TSH: thyroid-stimulating hormone; Anti-TG: anti-thyroglobulin antibody; Anti-TPO: anti-thyroid peroxidase antibody; CEA: carcinoembryonic antigen; TT: total thyroidectomy; CLND: central lymph node dissection; LLND: lateral lymph node dissection.

**Table 2:** Histopathologic findings.

Variables	Sporadic Group (*n* = 66)	Hereditary Group (*n* = 11)	*p*
Tumor size (mm), Median (IQR)	13.0 (7.0–22.2)	13.0 (5.0–20.0)	0.749
Variants, *n* (%)			
Isolated	47 (71.2)	10 (90.9)	0.581 ^a^
Composite	4 (6.1)	0 (0.0)	
Collision	15 (22.7)	1 (9.1)	
Right-lobe localization, *n* (%)	36 (54.5)	6 (54.5)	0.999 ^b^
Left-lobe localization, *n* (%)	28 (42.4)	5 (45.5)	0.999 ^a^
Isthmus localization, *n* (%)	3 (4.5)	0 (0.0)	0.999 ^a^
Multicentricity, *n* (%)	2 (3.0)	4 (36.4)	**0.003 ^a^**
Extrathyroidal extension, *n* (%)	9 (9.1)	3 (27.3)	0.113 ^a^
Surgical margin positivity, *n* (%)	5 (7.6)	2 (18.2)	0.261 ^a^
Capsular invasion, *n* (%)	7 (10.6)	2 (18.2)	0.608 ^a^
Vascular invasion, *n* (%)	20 (30.3)	4 (36.4)	0.732 ^a^
Neural invasion, *n* (%)	3 (4.5)	1 (9.1)	0.467 ^a^
Concurrent PTC, *n* (%)	17 (25.8)	1 (9.1)	0.441 ^a^

^a^ Mann–Whitney U test was used. ^b^ Pearson Chi-square test was used. Note: IQR: inter-quartile range.

**Table 3:** TNM prognostic classification.

Variables		Sporadic Group (*n* = 66)	Hereditary Group (*n* = 11)
TNM	T_1a_N_0_M_0_	27 (40.9)	6 (54.5)
	T_1a_N_1a_M_0_	1 (1.5)	0 (0.0)
	T_1a_N_1b_M_0_	2 (3.0)	0 (0.0)
	T_1b_N_0_M_0_	11 (16.7)	0 (0.0)
	T_1b_N_1a_M_0_	2 (3.0)	1 (9.1)
	T_1b_N_1b_M_0_	4 (6.1)	2 (18.2)
	T_2_N_0_M_0_	4 (6.1)	0 (0.0)
	T_2_N_1b_M_0_	7 (10.6)	2 (18.2)
	T_3a_N_0_M_0_	2 (3.0)	0 (0.0)
	T_3a_N_1b_M_0_	2 (3.0)	0 (0.0)
	T_3_N_0_M_0_	1 (1.5)	0 (0.0)
	T_3_N_1b_M_0_	1 (1.5)	0 (0.0)
	T_4a_N_1a_M_0_	1 (1.5)	0 (0.0)
	T_4a_N_1b_M_0_	1 (1.5)	0 (0.0)
Anatomical stage, *n* (%)	1	38 (57.6)	6 (54.5)
	2	7 (10.6)	0 (0.0)
	3	3 (4.5)	1 (9.1)
	4A	18 (27.3)	4 (36.4)
Pathological stage, *n* (%)	1	36 (54.5)	6 (54.5)
	2	6 (9.1)	0 (0.0)
	3	9 (13.6)	1 (9.1)
	4	15 (22.7)	4 (36.4)

TNM: tumor–node–metastasis system.

**Table 4:** Clinical features of the index nodules.

Variables	Sporadic Group (*n* = 69)	Hereditary Group (*n* = 11)	*p*
Volume (cc), Median (IQR)	1.20 (0.30–5.85)	1.00 (0.20–5.00)	0.660
Longitudinal diameter (mm), Median (IQR)	16.0 (10.0–26.6)	15.0 (10.6–24.0)	0.706
AP/T	0.82 (0.70–0.90)	0.77 (0.66–0.85)	0.360
Axial localization, *n* (%)			
Right	35 (50.7)	4 (36.4)	0.584 ^a^
Left	31 (44.9)	7 (63.6)	
Isthmus	3 (4.3)	0 (0.0)	
Longitudinal localization, *n* (%)			
Upper third	8 (11.6)	2 (18.2)	0.745 ^a^
Upper two-thirds	2 (2.9)	0 (0.0)	
Middle third	33 (47.8)	6 (54.5)	
Inferior third	5 (7.2)	1 (9.1)	
Lower two-thirds	10 (14.5)	2 (18.2)	
Completely	11 (15.9)	0 (0.0)	
Echogenicity, *n* (%)			
Hypoechoic	26 (37.7)	2 (18.2)	0.276 ^a^
Isoechoic	22 (31.9)	3 (27.3)	
Iso-hypoechoic	21 (30.4)	6 (54.5)	
Calcification, *n* (%)			
No	31 (44.9)	7 (63.6)	0.742 ^a^
Macro	15 (21.7)	2 (18.2)	
Micro	16 (23.2)	1 (9.1)	
Micro and macro	7 (10.1)	1 (9.1)	
Halo sign, *n* (%)	7 (10.1)	0 (0.0)	0.585 ^a^
EU-TIRADS			
3	16 (23.2)	4 (36.4)	0.377 ^a^
4	19 (27.5)	4 (36.4)	
5	34 (49.3)	3 (27.3)	
BETHESDA			
1	0 (0.0)	3 (27.3)	**0.018 ^a^**
2	3 (4.3)	0 (0.0)	
3	3 (4.3)	0 (0.0)	
4	2 (2.9)	0 (0.0)	
5	22 (31.9)	4 (36.4)	
6	39 (56.5)	4 (36.4)	

^a^ Mann–Whitney U test was used. Note: IQR: inter-quartile range; EU-TIRADS: European Thyroid Imaging Reporting and Data System; BETHESDA: Bethesda System for Reporting Thyroid Cytopathology.

**Table 5:** Clinical features of the patients with concurrent PTC.

Variables	Sporadic Group (*n* = 17)	Hereditary Group (*n* = 1)	*p*
PTC variants			
Classic, *n* (%)	14 (82.4)	1 (100.0)	0.999 ^a^
Follicular, *n* (%)	5 (29.4)	0 (0.0)	0.999 ^a^
Tall cell, *n* (%)	1 (5.9)	0 (0.0)	0.999 ^a^
Warthin-like, *n* (%)	1 (5.9)	0 (0.0)	0.999 ^a^
PTC localization			
Right, *n* (%)	10 (58.8)	0 (0.0)	0.444 ^a^
Left, *n* (%)	8 (47.1)	1 (100.0)	0.999 ^a^
Isthmus, *n* (%)	4 (23.5)	0 (0.0)	0.999 ^a^
PTC diameter (mm), Median (IQR)	7.0 (3.0–13.5)	1.0 (n.a.-n.a.)	0.111

^a^ Fisher’s exact test was used. Note: PTC: papillary thyroid carcinoma; IQR: inter-quartile range.

## Data Availability

The datasets generated and/or analyzed during the current study are not publicly available but are available from the corresponding author on reasonable request.

## Supplementary Materials

The following supporting information can be downloaded at: https://www.mdpi.com/article/10.3390/medicina61071164/s1, Table S1: Variants identified in patients undergoing genetic analysis.
