# Supplementary material for: A Comparative Study on the Multidimensional Features of Hereditary and Sporadic Medullary Thyroid Carcinoma Patients: A Single-Center Retrospective Study
Source: Medicina (Kaunas). 2025 Jun 27;61(7):1164. doi: 10.3390/medicina61071164 (PMC12299098; doi:10.3390/medicina61071164)
Supplement: Supplementary file 1 [file medicina-61-01164-s001.zip › medicina-3700531-supplementary.pdf]

Table S1. Variants identified in patients undergoing genetic analysis

Exon	Gene	Nucleotide change	Amino acid change	Mutation type	ACMG classification	Number of cases
11	*RET*	c.1901G>A	p.Cys634Tyr	Heterozygous missense mutation	Pathogenic (associated with MEN2A)	4
14	*RET*	c.2410G>A	p.Val804Met	Heterozygous missense mutation	Likely pathogenic (associated with FMTK)	4
15	*RET*	c.2712C>T	p.Ser904=	Synonymous polymorphism	Likely benign (FMTK-associated)	1
15	*RET*	c.2692_2703del	p.Asp898_Glu901del	In-frame deletion	Variant of uncertain significance	1
19	*RET*	c.3116C>A	p.Pro1039Gln	Missense variant	Variant of uncertain significance	1

RET: rearranged during transfection (RET) proto-oncogene.
